# Scanning electron microscopic analysis of adherent bacterial biofilms associated with peri‐implantitis

**DOI:** 10.1002/cre2.741

**Published:** 2023-05-08

**Authors:** Jae W. Chang, Jiarui Bi, Gethin Owen, Ya Shen, Markus Haapasalo, Colin Wiebe, Rana Tarzemany, Hannu Larjava

**Affiliations:** ^1^ Division of Periodontics, Faculty of Dentistry University of British Columbia Vancouver British Columbia Canada

**Keywords:** bacteria, biofilm, peri‐implantitis

## Abstract

**Objectives:**

Peri‐implantitis (PI) is caused by bacteria in the peri‐implant space but the consensus on microbial profile is still lacking. Current microbial sampling of PI lesions has largely focused on analyzing bacterial species that have been shed from the implant surface and captured in the pocket fluid. The purpose of the present study was to investigate the morphotypes of bacteria in biofilm covering the implant threads and explore whether certain morphotypes were associated with PI.

**Methods:**

Fourteen failed implants were removed and instantly processed for scanning electron microscope analysis. The implants were imaged at three equally divided sub‐crestal levels of the exposed area. Bacterial morphotypes were identified and quantified by three examiners. Mobility and years in function were correlated to the presence of different morphotypes.

**Results:**

The implants demonstrated the presence of variable bacterial morphotypes that did not correlate to disease progression in our study. Some implants were dominated by filaments and others showed the presence of combinations of cocci/rods or spirilles/spirochetes. In general, all implants showed variable morphologic biofilm composition. However, individual implants tended to have similar composition throughout the entire implant. Rods and filaments were dominant morphotypes throughout the surfaces and cocci showed increased presence toward the apical third. There were some differences in the biofilm morphology with mobility and time in function.

**Conclusions:**

The profiles of bacterial biofilm morphotypes in failing implants with similar clinical presentations were highly variable. While there were significant differences between implants, similar morphotypes in individual implants were often found throughout the entire surface.

## 1 INTRODUCTION

Peri‐implantitis is a biofilm initiated infectious condition that causes bone loss around dental implants that can progress quickly and lead to implant failure (Daubert & Weinstein, 2019; Tonetti et al., 1995; Zheng et al., 2015). About 20% of individuals receiving dental implants develop peri‐implantitis that has many characteristics similar to periodontitis (periodontal disease) including inflammation and bone loss (Derks & Tomasi, 2015). In the context of periodontal disease, the current understanding of the role of biofilm points to the “keystone” pathogens and dysbiosis (Hajishengallis & Lamont, 2012; Lamont & Hajishengallis, 2015). According to this concept, a specific pathogen (or pathogens) drives the development of the biofilm to favor more pathogenic over‐protective commensal bacteria in a special environment and in interaction with the host (N. Yu & Van Dyke, 2020). While the peri‐implant and peri‐implantitis microbiomes remain to be further established, they are generally believed to be similar to those of periodontitis but have less diversity due to their special environmental niche (Berglundh et al., 2011). Specific bacteria present in peri‐implantitis lesions have been studied by extracting the biofilm from peri‐implant pockets and subjected to molecular biological analysis, often by 16S RNA sequencing (Padial‐Molina et al., 2016). In general, the peri‐implantitis microbiome differs from the periodontitis microbiome and represents a microbiologically distinct ecosystem (Dabdoub et al., 2013; Kumar et al., 2012). The overall characteristics of the microbial shifts from health show increased levels of anaerobic, gram‐negative, and motile bacteria in peri‐implantitis (Al‐Ahmad et al., 2018; Apatzidou et al., 2017; Kumar et al., 2012; Mombelli & Décaillet, 2011; Sanz‐Martin et al., 2017). In addition, the majority of studies report that peri‐implantitis sites exhibit a greater number of red and orange complex bacteria, such as *Treponema denticola*, *Tannerella forsythia*, *Porphyromonas gingivalis*, and *Fusobacterium nucleatum* (Belibasakis & Manoil, 2021; Korsch et al., 2021; McHugh, 2012; Sanz‐Martin et al., 2017; Schaumann et al., 2014; X. L. Yu et al., 2019). Several additional genera have been associated with peri‐implantitis, including *Desulfobulbus, Dialister, Filifactor, Fusobacterium, Mitsuokella, Porphyromonas, Anaerococcus, Anaerovorax, Anaerofilum, Exiguobacterium, Burkholderia, Staphylococcus, and Treponema* (Dabdoub et al., 2013; Kumar et al., 2012).

Less attention has been paid to biofilm analysis in its native form on failing implants. In fact, there are no comprehensive studies demonstrating the biofilm composition of extracted dental implants. The aim of the present study was to describe using high resolution scanning electron microscope the distribution of bacterial morphotypes on the failed implants and evaluate the association of different morphotypes to implant topography and clinical findings.

## 2 MATERIALS AND METHODS

### 2.1 Ethics

The study was approved by the Clinical Research Ethics Board at the University of British Columbia for all procedures involving clinical implant samples (protocol #H15‐01881). Informed written consent was obtained from the donors according to the Declaration of Helsinki.

### 2.2 Sample collection and chemical preparation

The specimens were collected at the Graduate Periodontics Clinic in the University of British Columbia, Vancouver, Canada, during routine patient care (University of British Columbia ethics protocol #H15‐01881). Implants removed due to peri‐implantitis were placed in physiological saline solution (0.9% NaCl) and immediately transported to the laboratory. The biofilms on failed dental implants were fixed with 2.5% glutaldehyde (EM grade, Electron Microscopy Sciences [EMS]), in 0.1M PIPES (piperazine‐N,N′‐bis (2‐ethanesulfonic acid) buffer (pH 7.4) (MP Biomedicals) for 30 min at room temperature. After fixation, the specimens were rinsed with 0.1M PIPES buffer solution three times to remove any unreacted glutaraldehyde. The specimens were then transported to the UBC Centre for High‐Throughput Phenogenomics for processing and imaging. First, the samples were postfixed in 1% osmium tetroxide in 0.1M PIPES pH 6.8 for 1 h then rinsed thoroughly in double distilled water. Following postfixation, the samples were dehydrated in a graded series of ethanol (EtOH EM Grade: EMS) for 5 min each at 50%, 60%, 70%, 80%, 90%, and three times 5 min at 100%.

After dehydration, the samples were processed through critical point drier using liquid carbon dioxide as a transition fluid (Tousimis Research Corporation). Once fixed, dehydrated, and dried. Two samples were mounted onto aluminum stubs with epoxy glue and the remaining 12 samples were secured onto a large set screw vice stub (Ted Pella Inc), allowing two sides to be analyzed by scanning electron microscopy. Samples were then sputter‐coated with 20 nm of iridium using the Leica EM MED020 Coating System (Leica Microsystems).

### 2.3 Scanning electron microscopy imaging

Each sample was examined using a scanning electron microscope (Helios NanoLab 650 Focused Ion Beam SEM, Thermo Fisher Scientific). The implant samples were imaged in low voltage conditions (1 keV; secondary electron mode) for true surface imaging, under various magnifications from 65x to 50,000x throughout the investigation. However, the study images of samples were taken at the magnification of 5000x for the sole purpose of morphotype quantification.

Scanning electron microscopy acquired images in 16‐bit gray scale were manually pseudo‐colored using Adobe Photoshop (Adobe Photoshop Version 22.4.2). The artificial colors were applied to gray scaled images as a way of enhancing images for visualization and in this case outlining different morphotypes.

### 2.4 Quantification of morphotypes

The biofilm images were acquired at different levels in relation to the apico‐coronal location of the bone loss on each specimen. The level of bone loss was determined from the radiograph using the most advanced mesial or distal site. Each implant fixture was imaged at three levels of bone loss (coronal, central, and apical) and from two opposite sides. At each location, three random 5000x magnification images (with a horizontal field width of approximately 30 microns and pixel resolution of approximately 20 nm) were taken, resulting in a total of 18 images from each implant (3 levels x 2 sides x 3 images = 18 images). Each image was used for quantification of the percentage of main morphotypes (i.e., cocci, rods, filaments, and spirilla/spirochetes) by all three examiners.

### 2.5 Statistical analysis

The independent variables were bacterial morphotypes. Three examiners received the images for determination of visual estimates of percentages of the four main bacterial morphotypes. To assess the inter‐rater reliability, the first three samples were scored by the three investigators and the inter‐rater reliability test was performed using Cohen's kappa testing in the IBM SPSS version 27.0 software for Mac (SPSS Inc.). With a kappa value >0.6, it is acceptable to proceed with the rest of the samples that were scored by all three examiners (McHugh, 2012). The scores for each image were averaged to one number (%) for each of the morphotypes. Therefore, at any level the six images received the mean number resulting in six replicate measurements for statistical purposes. The quantification data were compiled in Microsoft Excel (version 16.49) according to the examiners and samples. The statistical analyses included descriptive statistics (mean ± standard error of the mean) for each sample and the Mann–Whitney U test was used to compare two population means for independent variable of, “Mobility [reported clinical mobility or 100% bone loss]” and “Years in function [10 years and up versus less than 10 years]” with significance level of *p* < .05. Bacteria proportion (%) was a numerical variable that the present study analyzed to compare between subgroups (three locations or four morphotypes). Since there were more than two subgroups, one‐way analysis of variance (ANOVA) was performed which shows the comparison of mean values between subgroups and showed if overall difference existed when *p*‐value was less than 0.05. Then, a post‐hoc test for pair‐wise comparison between all subgroups and Tukey's HSD was chosen to determine which subgroups were different.

## 3 RESULTS

### 3.1 Clinical and radiographical findings

**Figure 1:**
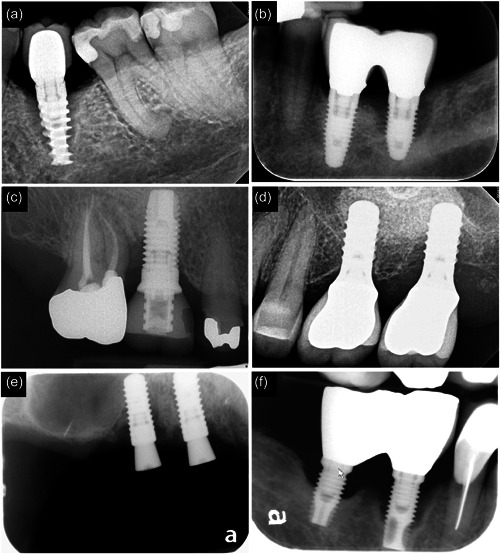
Radiographic images of a set of implants removed due to severe bone loss. These implants represent different thread type (aggressive [a], regular [b, c, e, f] or mild [d]), surface characteristics (oxidized [a–c, f] or SLA [d, e]), level of placement (bone [a–c, e, f] or tissue level [d]) and abutment fit (butt joint [b–d, f] or platform‐shifting [a, e]) and splinted (b and f) and nonsplinted (d and e) adjacent implants. SLA, Sand‐blasted, large grit, acid‐ etched.

A total of 14 failed implants were analyzed for the present study and the examples of their radiographs are illustrated in Figure 1. The mean time in function for the implants was 7.9 years with a range of 2–15 years. The majority of these implants had anodized surface (*N* = 11, Nobel Biocare). The amount of radiographic bone loss varied between 47.1% and 100% and five implants were mobile at the time of removal. Out of the 234 images in total, 213 images were analyzed, and 21 images (9%) were excluded. The excluded images were not quantifiable due to poor sample processing, poor image quality or lack of clearly identifiable biofilm at a 5000X magnification. In addition, images of highly heterogeneous microbiomes were captured at variable magnifications for illustrative purposes.

### 3.2 Differences in morphotypes in different locations

**Figure 2:**
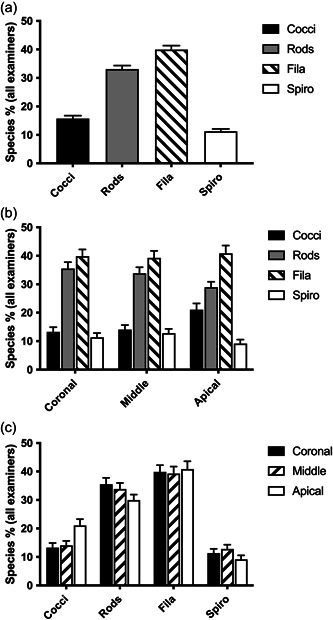
Relative distributions of each morphotype in each location and all combined surfaces. (a) Relative distribution of each microbiome morphotype combined with all samples. (b) Relative distribution of each morphotype combined with all samples in each location of implants. (c) Relative distribution of morphotypes at each implant location combined with all samples. *N* = 14.

Overall rods and filaments dominated all the surfaces of the failed implants with a combined load of 70%–75% of all morphotypes, regardless of the location (Figure 2). Cocci (13%–21%) and spirochetes/spirilla (9%–13%) composed the rest of the morphotypes. Interestingly, cocci showed a higher abundance in the apical level compared to more coronal sites (Figure 2). However, filament morphotype was the most dominant species in apical location (40.86%). Overall and surprisingly, the variation of morphotypes at different levels of individual implants was low, that is, each implant often possessed similar morphotypes throughout the exposed threads from coronal to apical locations (Supporting Information: Figures 1 and 2). Only one implant showed significant colonization with spirochetes/spirilla (32%–57%). Overall, the colonization by spirochetes/spirilla was relatively low through the specimen population (Figure 2). The results analyzed with one‐way ANOVA showed that rods, filaments, and spirilla/spirochetes morphotypes were not significantly different in the different locations.

### 3.3 Association of morphotypes with mobility

**Figure 3:**
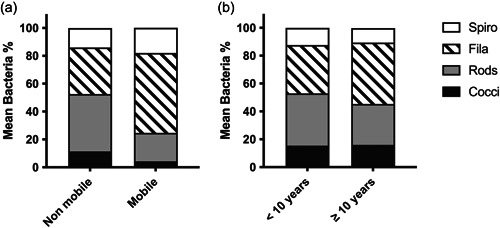
Relative distribution of morphotypes related to implant mobility, and more or less than 10 years of function. (a) Relative distribution of morphotypes in mobile and nonmobile implants. Mann–Whitney test, *p* < .05 between nonmobile versus mobile on rods, filaments, and spirochete/spirilla. *N* (nonmobile) = 9, *N* (mobile) = 5. (b) Relative distribution of morphotypes in Years in function, <10 years versus ≥10 years. Mann–Whitney test, *p* < .05 between <10 years versus 10 years and up on rods, filaments, and spirochete/spirilla. *N* (<10 years) = 7, *N* (10 years and up) = 7.

The sample size for nonmobile implants group and mobile implants group were 9 and 5. The average years in function for the implants that were mobile at the time of explantation was 7.2 years and that of the nonmobile implant was 8.3 years. Average bone loss on the mobile implant group was 100% by definition and 67% for those implants that were still firm at the time of explantation. These two different groups of implants had statistically significant differences in rods, filaments, and spirochetes/spirilla morphotypes (Figure 3a). The dominant morphotypes of nonmobile implants were rods (41% vs. 21%) while mobile implants were dominated by filaments (49% vs. 34%; Figure 3a). Spirochetes/Spirilla were higher in the nonmobile implants over mobile implants at 14% and 7%, respectively (Figure 3a).

### 3.4 Association of morphotypes with years in function with 10‐year cut‐off

The average years in function for the group of implants that had less than 10 years in function was 4.6. The other group of 10 and more years in function had an average year in function of 11.3 years. The sample size was seven implants in each group. Average bone loss on the “less‐than‐10 years” and “10 and more years” groups were 81.3% and 76.4%, respectively. The abundance of cocci and spirochetes/spirilla was similar in both groups (Figure 3b). The dominant morphotypes of the “less‐than‐10 years” group were rods (37.5%) while filaments (44.0%) were the most prevalent morphotype for the “10 years and more” group, respectively (Figure 3b).

### 3.5 Images of biofilm on the surfaces of the failed implants

**Figure 4:**
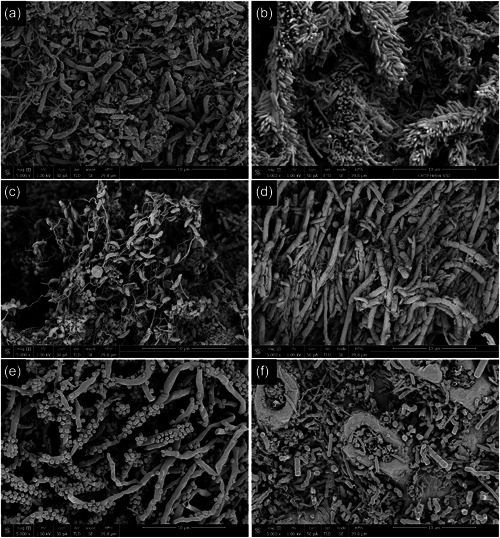
High resolution scanning electron microscopy images on the surfaces of the failed implants. (a, b) The images of the coronal third surfaces of failed implants show abundant rods and spirochetes (a) and bristle‐brush formations (b); (c, d) the images of the middle third surface of failed implants show curved rods (vibrios) with flagellas (c) and rods and filaments (d); (e, f) the images of the apical third surfaces of failed implants demonstrate the presence of corn‐cob formations with central filament coaggregating with cocci (e) and rods and filaments (f). Images were in 5000x magnification (magnification bar included in each image).

**Figure 5:**
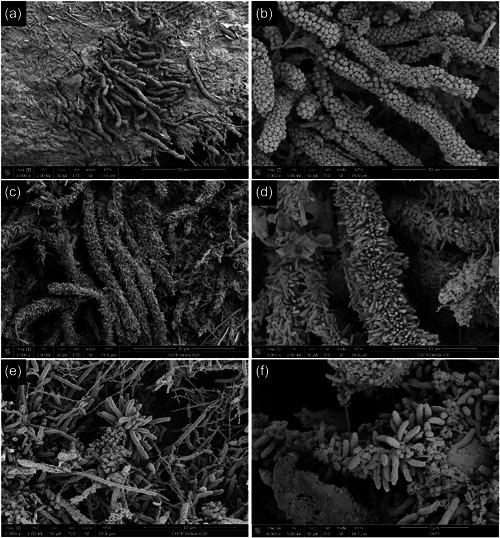
Coaggregate structures of the biofilm on the surfaces of failed implants. (a, b) Corncobs on the coronal third areas of failed implants (a, 1000x; b, 5000x); (c, d) test‐tube brush formations on the middle third areas of failed implants (c, 2000x; d, 5000x); (e, f) hedgehog structures on the middle third areas of failed implants (e, 5000x; f, 8000x).

**Figure 6:**
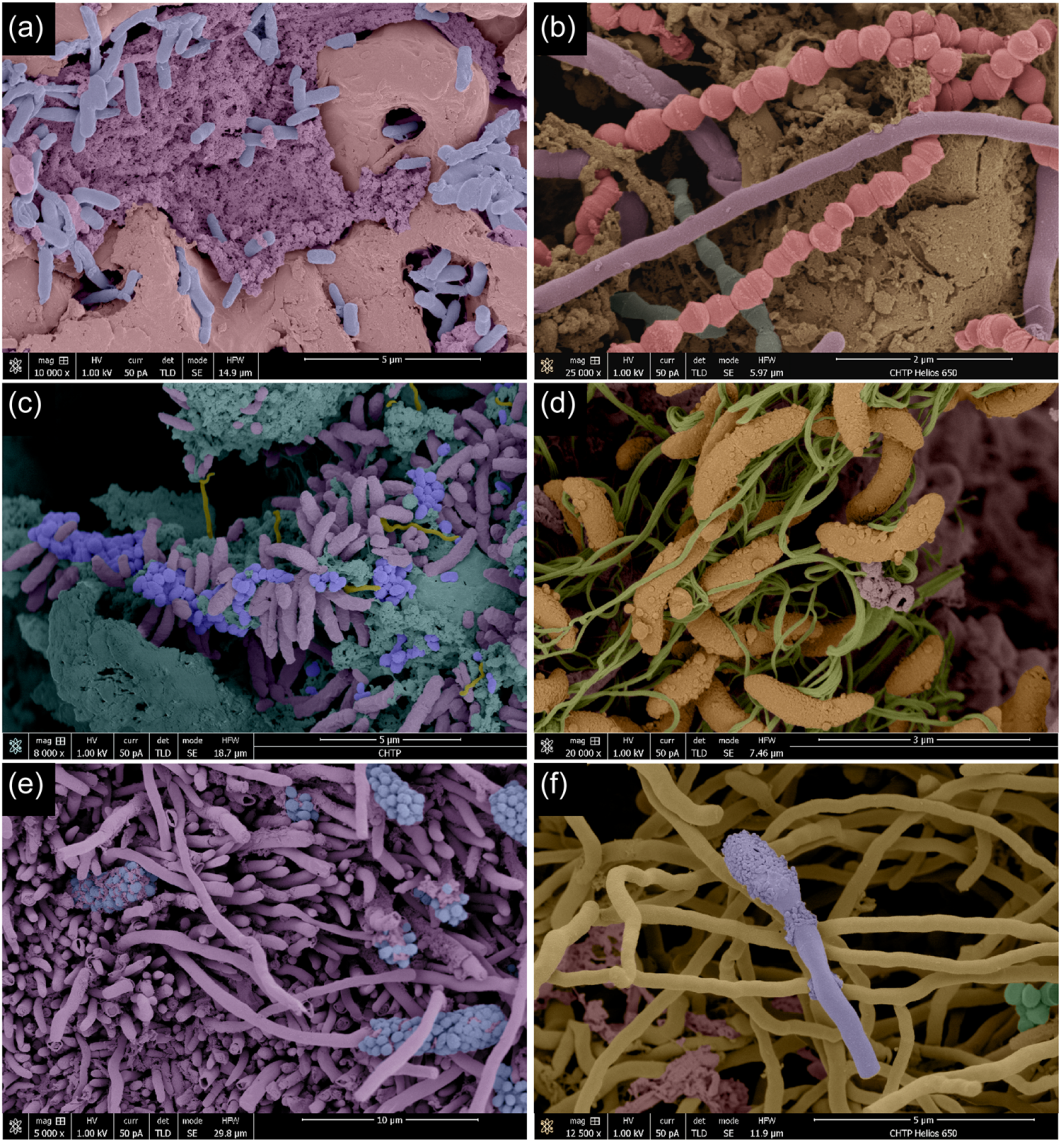
Pseudo‐colored scanning electron microscope images of native biofilm on the surfaces of the failed implants. (a) Rods (blue) around the pores of the anodized implant surfaces (pink; 10,000x). The matrix is colored purple. (b) Cocci (pink) along with filaments (purple) embedded in matrix (brown) (25,000x). (c) Hedgehog formation with cocci (blue), rods (purple) around matrix (green) (8000x). (d) Morphotype of vibrio (curved rods, orange/brown) covered with vesicles and elongated fimbriae/flagella (green) (20,000x). (e) Multiple strands of the corncob formation with filaments (purple) and cocci (blue) (5000x). (f) Unknown morphotype (purple) with filamentous morphotypes (yellow), cocci (green), and matrix (pink) (12,500x).

In the present investigation, examples of individual biofilms present on failed dental implants are illustrated in Figures 4, 5, 6 and Supplemental Figures 1 and 2. Even though high‐level heterogeneity of biofilm with the various morphotypes of bacteria was common (Figure 4), rods and filaments were the dominant morphotype in all failed implants. The early stages of corncobs were captured with the filaments slowly being occupied by cocci. At higher magnifications, the images showed more intimate contact among various species. These high‐resolution images illustrated different levels of heterogeneity with communication, formation, and diversity. There were abundant matrix layers around or on top of bacteria and complex structures were often associated between bacteria. Coaggregation of bacterial species were often present, representing typical “corncobs” (Figure 5a,b) and “test tube‐brush” (Figure 5c,d) formations with central filament covered either by cocci or rods, respectively. “Hedgehog” formations were evident, but they were scarcer than corncobs and test tube brush complexes (Figure 5e,f).

A set of biofilm images were pseudo‐colored for a better visual examination. Using images captured with high magnifications (usually more than 10,000X), different species and contrasting backgrounds and matrix were colored for better visualization. Some of the rods (blue) were identified easily on relatively bare surfaces of the implant (pink) with granular matrix (purple) (Figure 6a).

Also utilizing different color schemes, it was easy to illustrate the cocci‐like morphotypes (red) from filaments (purple) (Figure 6b), rods (purple), cocci (blue), and matrix (green) (Figure 6c), curved rods (vibrios; orange) with surface vesicles and multiple flagellas (green) (Figure 6d), early stage of corncob formation with central filament (purple) interacting with cocci (blue) (Figure 6e) and long filaments (beige) with long rod with enlarged “head” with vesicles (purple) (Figure 6f).

## 4 DISCUSSION

The present study investigated native adherent biofilms on failing implants to map the morphological characteristics of biofilms associated with peri‐implantitis. Surprisingly, there are no high‐resolution images published previously on failing implants. The main findings of the present study indicate that the biofilm covering failing dental implants is dominated by rods and filaments and lesser degree by cocci and spirochetes. Interindividual differences were, however, high and biofilm surface morphology appeared to be unique for each failed implant. In addition, many of the implants appeared to have similar morphotypes present throughout the implant surface. Furthermore, several failed implants contained biofilm that morphologically gave an impression of infections by a few species. These findings agree with previous research performed by sequencing that reveal lower diversity in peri‐implantitis biofilm (Berglundh et al., 2011). Several studies have also reported high variation of species between individuals (Korsch et al., 2021; Schaumann et al., 2014; X. L. Yu et al., 2019). In general, new generation sequencing studies have demonstrated that peri‐implant sites are distinct ecological niches and harbor different microbiome in health and disease from natural teeth. However, no clear consensus exists regarding the specific microbiota in peri‐implantitis (reviewed in Belibasakis & Manoil, 2021).

The present study to characterize biofilm on failing dental implants has similarities to pioneering work by Listgarten (1976) who described the different morphotypes in periodontitis lesions. Listgarten (1976) studied the bacterial flora on the extracted teeth and described the morphotypes of bacteria of various clinical stages such as health, gingivitis, periodontitis, periodontosis, and postperiodontosis. The distinct morphotypes were noted, including coccoid species in the healthy sites and an increased filamentous types evident in the inflamed gingival tissues. In 3‐week‐old biofilm, the filaments were dominant, and the cocci were scarce. After 3 weeks, there was no further morphological evolvement except the free‐floating spirochetes layers evident on the most superficial layer. The authors also reported the presence of corncobs‐like structures as complexes consisted of filaments surrounded by cocci. Also, the corncobs were described being on the surface of this bacterial mass and speculated to represent a transition to anaerobic flora with spirochetes as the severity of disease worsened. The current investigation witnessed similar features in the peri‐implantitis biofilm, including frequent corncob formations that sometimes dominated along the entire implant surface. However, spirochetes appeared to be less frequent than in periodontitis biofilm described by Listgarten. The dominance of *Spirochetes* in periodontitis pockets have been demonstrated in multiple other studies as well (Listgarten & Hellden, 1978; Mark Welch et al., 2016). This observation could be for multiple reasons. The pocket environment at the deepest sites of peri‐implantitis has different immunological environment, including more acute phase cells and no separation of bacteria from inflammatory lesion by pocket epithelium (Moon et al., 1999). This difference to chronic periodontitis could perhaps lead to more efficient clearance of motile spirochetes. However, there could be multiple other reasons for low presence of *Spirochetes*. *Spirochetes* are motile species (Li et al., 2000) and they maybe under‐presented in the attached biofilm and present more abundantly in the unattached peri‐implantitis pocket. Furthermore, the quantification method used in the present study could underestimate their relatively proportion as they are typically smaller in size than the other bacteria in the peri‐implantitis biofilm.

Only a few studies have identified different species of bacterial in native biofilm on teeth and none on biofilms on failing implants. Zijnge et al. (2010) showed the biofilm on the natural teeth and analyzed the architecture of the biofilm using Fluorescent In Situ Hybridization (FISH) technique (Zijnge et al., 2010). In subgingival biofilm investigation, the authors reported that there were four different distinct layers depending on the bacterial morphotypes and intensities of fluorescence. The first layer showed only the *Actinomyces* sp. that have rod‐shape as individual bacteria. The intermediate layer was reported to have *F. nucleatum (rod), T. forsythia (rod)* and *Tannerella* sp. (*rod*). Common species in the outer layers of biofilm and intermediate layers were “*Cytophaga‐Flavobacterium‐Bacteroides* cluster” as mixture of filamentous, rods‐shaped, or occasional cocci‐shaped bacteria. Interestingly, the authors called the *Synergistetes* group A bacteria, a large “cigar‐like bacteria” in the superficial layer depicting them in a “palisade” arrangement. A fourth layer was described as a loose layer with *Spirochetes* as a primary species. In these most superficial layers, the authors were able to detect the complexes of multispecies structures such as test‐tube brushes. Ever since the test‐tube and corncobs were described (Listgarten, 1976), this study was the first study to identify the species associated with those complexes. The authors were able to identify those species forming the “test‐tube” brushes as *T. forsythia, Campylobacter* sp., *Parvimonas micra*, *Fusobacteria* and *Synergistetes* group. The core structures were consisted of *T. forsythia, F. nucleatum*, and central axis of yeast cells or hyphae. The author's conclusion was congruent to the findings of Kolenbrander and London (1993) that reported *F. nucleatum* evident in the intermediate layer. Mark Welch et al. (2016) reported the biogeographical analysis on supra‐ and subgingival plaque using the same FISH technique. In their analysis of multigenus consortia, physical structures of filamentous species extending from the base and peripheral areas of extensions were covered by cocci. In that context, these co‐aggregates appear to be a part of even larger superstructure, named hedgehog structures where *Corynebacterium* forms the foundation with long filaments extending through the entire biofilm aggregate (Mark Welch et al., 2016). Toward the surface of this multigenus consortium, the tips of *Corynebacterium* filaments are coated by cocci or rods forming corncob‐like structures. In that study, the corncobs had either single layer of *Streptococci* or *Porphyromonas* covering the *Corynebacterium* filament or double layer consisting of a combination of *Streptococci* as the inner layer and *Haemophilus/Aggregatibacter* as the outer layer (Mark Welch et al., 2016). The most common corncob had single layer of *Streptococci* and partial layer of *Haemophilus/Aggregatibacter* (Mark Welch et al., 2016). Previously, *F. nucleatum* has been shown to form corncobs in vitro but they were not commonly involved in these structures in supragingival plaque (Kolenbrander & London, 1993; Mark Welch et al., 2016). Nine taxa were common in forming the hedgehog complex: *Corynebacterium, Streptococcus, Porphyromonas, Haemophilus/Aggregatibacter, Neisseriaceae, Fusobacterium, Leptotrichia, Capnocytophaga, and Actinomyces*. The authors reported it was *Corynebacterium* that forms the core filamentous axis and *Streptococcus* cells (*Hemophilus/Aggregatibacter*, *Porphyromonas*) were at the distal tip creating this unique spatial arrangement (Mark Welch et al., 2016). The bacterial species forming the complex structures of peri‐implantitis biofilm observed in the present study are likely similar to those observed on teeth described above (Donos et al., 2012). However, this needs to be confirmed in future studies.

There are several limitations of this study including the process of sample collection, fixation timing of samples, quantification of morphotypes and accuracy of determining the bone loss areas. The method of removing a failed implant may vary and damage the surface. Therefore, the biofilms analyzed for the present study may not have been 100% representative. There might have been contamination on the sample or translocation of the biofilm within the sample. Processing of the specimens may have also caused artifacts and removed some of the biofilm. Determining bone loss using the radiographs may not have been completely accurate depending on the quality of radiograph. Last, the quantification system could have introduced errors.

## 5 CONCLUSION

Overall rods and filaments dominated all the surfaces of the failed implants while the abundance of spirochetes/spirilles was low. The current investigation showed heterogeneity in the biofilm bacterial morphotypes on surfaces of individual failed implants with similar clinical outcomes. Often, however, morphotypes were surprisingly similar throughout the surfaces of individual failed implants. Our observations suggest that individual diverse microbiomes could be associated with peri‐implantitis. The complex nature of native biofilm on implant surfaces on failed implants as observed in the present study presents a difficult task for decontamination and explains why peri‐implantitis treatment regimens often fail or are only partially successful. More studies are needed to identify individual species on native peri‐implantitis microbiome. In addition, novel techniques are needed to decontaminate native biofilms on implants that suffer from mild to moderate peri‐implantitis.

## AUTHOR CONTRIBUTIONS

All authors have made substantial contributions to the design of the study. Jae W. Chang, Colin Wiebe, and Rana Tarzemany collected the clinical data and the implants for the study. Jae W. Chang, Jiarui Bi, and Gethin Owen processed the samples and collected the images for analysis. Jae W. Chang, Ya Shen, Jiarui Bi, Markus Haapasalo, and Hannu Larjava analyzed and processed the data. Jae W. Chang, Jiarui Bi, and Hannu Larjava drafted the manuscript. Gethin Owen, Ya Shen, Markus Haapasalo, Colin Wiebe, and Rana Tarzemany critically revised the manuscript.

## CONFLICT OF INTEREST STATEMENT

The authors declare no conflict of interest.

## Supporting information

Supplemental Figure 1. Representative high‐resolution images of adherent biofilm on different locations (Coronal, Middle, and Apical) of failed implants. Samples 1–7 are shown with 5000x magnification.

Supplemental Figure 2. Representative high‐resolution images of adherent biofilm on different locations (Coronal, Middle, and Apical) of failed implants. Samples 7–14 are shown with 5000x magnification.

## Data Availability

The data that support the findings of this study are available from the corresponding author upon reasonable request.
